# Adaptive hybrid virtual inertia controller for PMSG-based wind turbine based on fuzzy logic control

**DOI:** 10.1038/s41598-025-87986-6

**Published:** 2025-01-30

**Authors:** Mohamed Hosny, Mostafa I. Marei, Ahmed M.I. Mohamad

**Affiliations:** Electrical Power and Machines Department, Faculty of Engineering, Ain Shams University, Ain Shams, Egypt

**Keywords:** Adaptive hybrid virtual inertia control, Frequency support, Virtual capacitance control, Low inertia systems, Microgrids, Electrical and electronic engineering, Energy science and technology, Renewable energy, Wind energy

## Abstract

The integration of renewable energy sources into microgrids presents some challenges due to the decreased system overall inertia associated with the presence of converter -based sources. To overcome this issue and to enhance the system inertia, various concepts for virtual inertial control have been proposed in the literature. However, the concept of improving the system frequency through wind turbines has gained widespread acceptance. Several frequency support techniques have been proposed recently. However, conventional virtual inertia controllers may not satisfy the performance requirements in terms of frequency nadir and rate of change of frequency (ROCOF) over a wide range of microgrid operating conditions. This paper proposes a hybrid adaptive virtual inertia control strategy based on Fuzzy logic. The hybrid strategy integrates kinetic energy based virtual inertia control and virtual capacitance control schemes. The gains of both KE based virtual inertia control loop and virtual capacitance control loop are adapted simultaneously to enhance frequency support of low inertia microgrid under wide range of renewable energy sources (RES) disturbances and load changes. Different case studies are simulated on MATLAB/ Simulink to evaluate the dynamic performance of the proposed adaptive hybrid virtual inertia strategy under different conditions.

## Introduction

In response to environmental concerns, climate changes, the energy gap and rapid economic growth, the focus has been on using renewable energy sources (RESs) such as wind energy, based on power electronics to generate electricity so that large microgrids provide a viable framework for integrating RES in utility grids. RES are essential components in modern microgrids; however, it is important to maintain the dynamic performance of the microgrid’s frequency and voltage within accepted limits. The high penetration level of converter-based RES causes considerable challenges; however, the most crucial one is the reduced overall system inertia, which directly affects the dynamics of the system frequency^1,2^.

To enhance the dynamic behavior of low-inertia systems, several concepts of virtual inertia control have been implemented recently^1–4^. One of the most popular methods is to allow wind turbine generators (WTGs) to participate in enhancing the system inertia. This can be realized by inserting an additional control loop to the wind generator power control loop to simulate the inertial response characteristics of a conventional synchronous generator (SG). Different configurations are suggested to set up WTGs for frequency support via utilizing the energy stored in the WTG system. Two popular approaches are employed to enhance the dynamics of low-inertia systems. The first approach is to make use of the stored kinetic energy (KE) in the wind generator rotor and the second one is based on the stored electrostatic energy in the DC-link capacitor interfacing the machine and the grid converters^5–8^.

Based on the stored kinetic energy and the wind generator’s rotor, three popular strategies have been proposed to provide frequency regulation in low-inertia grids^9–13^. The first strategy is based on the derivation control, where the rate of change of frequency (ROCOF) is used as input signal for the frequency regulation loop. In the second strategy, power-frequency droop control based on system frequency deviation is utilized. The third strategy is based on de-loading the wind generator to maintain a reserve margin by moving the wind turbine’s operating point from the maximum power point to a lower power level.

The second approach to provide virtual inertia is based on utilizing the electrostatic energy stored in the DC-link capacitor of the WTG^8,13,16^. Compared to KE based methods, utilizing the energy of the DC-link capacitor becomes better choice because it has no effect on the maximum power point tracking (MPPT) control. To release or absorb the electrostatic energy of the DC-link capacitor, a DC voltage-frequency droop control loop is integrated into the gird side converter (GSC) controller^13–16^.

This control strategy allows the DC-link voltage reference to be modified in response to changes in the low-inertia frequency. This action results in reducing the output power oscillations. However, the virtual inertia provided by the DC-link capacitor is quite low and insufficient to provide reliable frequency support during power system disturbances. To overcome this limitation, virtual capacitance control (VCC) technique is proposed^14,15^. The VCC technique gets a benefit from the DC-link voltage variation in the presence of DC voltage droop control. It is worth mentioning that the VCC is integrated with the machine side converter (MSC) control system to provide active power during frequency variations.

Based on an intensive literature review, it is worth mentioning that the inclusion of the virtual inertial control loop for permanent magnet synchronous generator (PMSG) based WT is an essential requirement for sustaining the power system stability. In many control strategies, the virtual inertia is set to a constant value regardless of the level of RES penetration or the magnitude of the disturbance^14,17–19^.

To emulate the inertial behavior of SGs, constant control gain or constant virtual inertia gains (settings) are often used to give the system more inertia and allow the system to react to disturbances at all levels. In^13,14,17,20–22^, using fixed virtual inertia gains of the conventional frequency regulation controller prevents the system from providing adequate performance under varying load disturbance. Moreover, conventional virtual inertia control systems may not be suitable for ensuring widely acceptable performance of microgrid operations. In^15,23–26^, the fixed value of the inertia constant for any frequency controller is obtained by using mathematical formulas. However, the virtual inertia value is still not adaptable. Previous issues have been addressed in^27–33^ by introducing the Fuzzy logic (FL) control. However, applying the concept of fuzzy logic control to adapt the gains of the virtual capacitance controller (VCC) and KE virtual inertia controller has never been investigated before and still missing in the literature.

To fill in this gap, this paper presents a hybrid adaptive virtual inertia control strategy based on fuzzy logic to fully utilize the stored kinetic energy and the stored electric energy in the DC-link capacitor. Fuzzy logic is a heuristic learning method that establishes a control system using specialized or professional knowledge and human understanding. It can deal with nonlinearity and uncertainty present in power system operation and control, while remaining simple and reliable^30,32^. Further, the fuzzy logic helps to model complex responses, which cannot be described using mathematical equations to obtain the direct relations between the system inputs and outputs^33–39^. In this work, two FL units are proposed to adapt the gains of the KE-based virtual control loop and the VCC loop to provide inertial support to a low-inertia grid, considering different system model parameters and variations. Based on the previous discussion, the contributions of this paper to the field can be summarized as follows:

- Presenting adaptive hybrid virtual inertia controller to enhance the dynamic performance of a low- inertia system.
- Proposing two fuzzy logic units to adapt the gains of the KE-based virtual control loop and the VCC loop to provide inertial support to a low-inertia grid, considering different system model parameters and variations.
- Investigating the dynamic performance of the proposed adaptive hybrid virtual inertia strategy.
- Presenting performance comparison with the fixed gain and no inertia controllers, under different system parameters variations.

The paper is organized as follows: Sect. 2 discusses the PMSG-based WT system model, and the simplified SG used for this study. Various techniques used for AC frequency dynamics improvements including K.E stored in rotor speed and DC link stored energy are introduced in Sect. 3. Section 4 introduces the proposed controller including the strategy and implementation. Simulation results are given to validate the proposed control strategy in Sect. 5. The conclusions are summarized in Sect. 6.

### **System model**

**Fig. 1:**
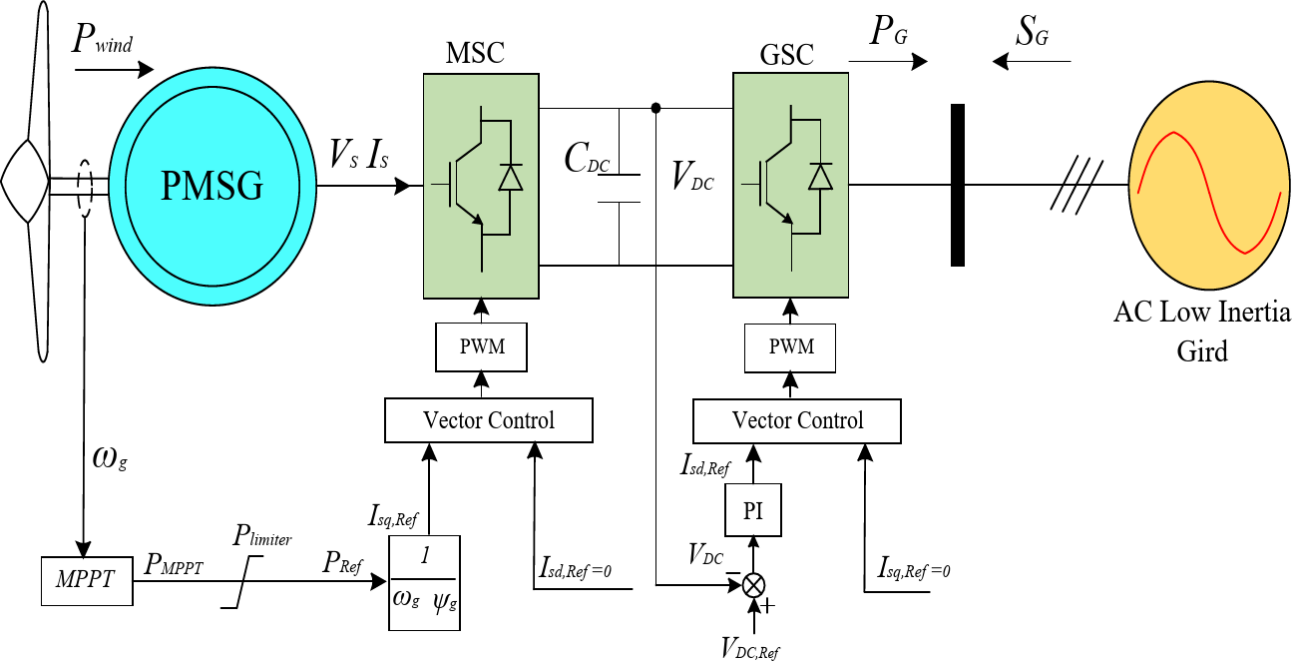
Block diagram of the PMSG-based wind turbine control.

Figure 1 shows the schematic diagram of the PMSG-based WT system connected to a low-inertia system. Through a compact back-to-back converter, the PMSG-based WT is interfaced to the AC side of the low -inertia system. By measuring the PMSG current *I*_*S*_ and voltage *V*_*S*_, the PMSG output electromagnetic torque *T*_*EM*_is represented in the rotating arbitrary synchronous frame as follows^40–42^:

1 $$\:{\text{T}}_{\text{EM}}\text{=1.5(}{\Psi}_{\text{m}}{\text{I}}_{\text{sq}}\text{+(}{\text{L}}_{\text{d}}\text{-}{\text{L}}_{\text{q}}\text{))}{\text{I}}_{\text{sd}}{\text{I}}_{\text{sq}}$$

where *I*_*sd*_ and *I*_*sq*_ are *dq* stator current components, *ψ*_*m*_ is the permanent magnet flux linkage, *L*_*d*_ and *L*_*q*_ are the stator windings inductance in the *d*- and *q*-axis, respectively. The rotor structure for PMSG is assumed to be symmetrical, in which *L*_*d*_ = *L*_*q*_. Therefore, Eq. (1) is simplified as follows:

2 $$\:{\text{T}}_{\text{EM}}\text{=1.5}\Psi_{\text{m}}{\text{I}}_{\text{sq}}\text{=}\frac{{\text{P}}_{\text{Ref}}}{{{\omega}}_{\text{g}}}\:$$

where *P*_*Ref*_is reference active power for the MSC. The objective of the GSC controller is to keep the DC-link voltage regulated to its reference value to guarantee the transfer of the active power supplied by the wind generator to the grid. The MSC plays an important role in controlling the generated active power, through regulating the shaft speed to achieve maximum power point tracking (MPPT)^43–47^. The mathematical description of the mechanical output power *P*_*wind*_ extracted from the wind is described by

3 $$\:{\text{P}}_{\text{wind}}=\:0.5\:\pi\:\rho\:{\text{R}}^{\text{2}}{\text{V}}_{\text{w}}^{\text{3}}{\text{C}}_{\text{p}}(\:\lambda,\beta\:)$$

4 $$\:{\lambda\:=\:}\frac{{{\omega}}_{\text{g}}\text{R}}{{\text{V}}_{\text{w}}}$$

where *ρ* is the air density, *R* is the blade rotor radius, *V*_*w*_ is the wind speed, *ω*_*g*_ is the rotor speed, *C*_*p*_ is the wind power coefficient which is a function of the tip speed ratio *λ*, and the pitch angle *β*.

The active power resulting from the MPPT technique is described as

5 $${\rm P_{MPPT}}=\frac{2\pi\:\rho\:C^{max}_{p}R^{5}}{\lambda^{3}}\omega^{3}_{g}=k_{opt}\omega_{g}3$$

where *k*_*opt*_ is the coefficient of the MPPT curve.

The simplified equivalent SG model is used to simulate the electromechanical performance of the low- inertia microgrid under different conditions. In^44,47^, an extensive mathematical formulation of the aggregated grid model is presented. In power systems, the frequency is controlled, firstly, by the system inertia and then followed by the primary and secondary frequency controllers. Therefore, the dynamics of the low-inertia grid are given by^14^:

6 $$\:{\Delta}{\text{P}}_{\text{E}}{\:=\:}-{\:}\frac{{2\:}{\text{H}}_{\text{eq}}{\text{S}}_{\text{G}}}{{\text{f}}_{\text{0}}^{\:\:2\:}}{f\:\:}\frac{\text{df}}{\text{dt}}$$

7 $$\:{\Delta}{\text{P}}_{\text{D}}{\:=\:-\:}\frac{{1}}{{{\rm R}}_{\text{eq}}}{\:\:\Delta\:f}$$

Where ∆*P*_*E*_ is the load or output electrical power, ∆*P*_*D*_ is output power by primary control, *R*_*eq*_ is droop factor, *H*_*eq*_ is the equivalent inertia of all connected SGs, SG is the rated MVA of the system, *f*_*0*_ is the nominal frequency, *∆f* is system frequency deviation and *f* is the instantaneous measured frequency of the microgrid.

The KE released or absorbed will cause the output electric power to increase or decrease, respectively. The inertial response of the SG produces an electromagnetic power proportional to the rate of change of frequency (ROCOF). For a microgrid, the allowable frequency deviation *∆f* is relatively low, ∆*f* = ± 1 Hz. As a result, “*f ≈"f*_*0*_”, and Eq. (6) is simplified to:

8 $$\:{\Delta}{{\rm P}}_{{\rm KE}}{\:=\:}\frac{{2\:}{{\rm H}}_{{eq}}}{{{\rm f}}_{{0}}^{{\:\:}}}{\:}\frac{\text{df}}{\text{dt}}$$

where ∆*P*_*KE*_ is the KE released or absorbed during the disturbance in per unit value. Therefore, it is noted that the term *H*_*eq*_ governs the frequency nadir and the ROCOF.

AC microgrids offer several advantages including compatibility with existing electrical infrastructure and ease of integration with traditional power sources. However, the integration of RES into AC microgrids presents unique challenges, particularly in maintaining frequency stability. Unlike conventional power systems which rely on large SGs with substantial mechanical inertia to regulate frequency, AC microgrids often incorporate power electronic-based RES that lack mechanical inertia. The absence of inertia can lead to significant frequency deviations, especially during sudden changes in load or generation.

Variations in frequency are caused by the power mismatch between the generation and the load during a disturbance event. The typical SG would therefore modify its power delivered to the AC grid to provide frequency support when this situation happens. In other words, during a frequency change, the KE stored in the SG’s rotor is rapidly changed.

## Virtual inertia techniques for frequency enhancment

### KE-based virtual inertia control loop

The KE-based virtual inertia control is achieved by adjusting the power output of PMSG wind turbines in response to grid frequency changes. The generated active power is dynamically modified by adding a power component to the MSC reference power command (P_MPPT_), which is proportional to system frequency variations. This additional active power is used as a supplement to the reference power supplied by the MSC during system transients. Due to the increase in generated active power, the PMSG will slow down, and the KE stored in the wind turbine rotating blades is released. Hence, the control loop simulates the inertial response of a conventional SG to effectively smooth out frequency fluctuations. The virtual inertia control loop, based on the KE of WT, takes different configurations to regulate frequency.

fluctuations. For the derivation method, the injected active power component to the MSC power loop, ∆*P*_*vir*_, is calculated as follows:

9 $$\:{\Delta}\:{{\rm P}}_{{\rm Vir}}{\:=\:2}{{\rm \:H}}_{{\rm G}}{\:\:f\:}\frac{\text{df}}{\text{dt}}$$

10 $$\:{\text{H}}_{\text{G}}{\:=\:}\frac{\text{J}{{\omega}}_{\text{g}}^{{\:}\text{2}}}{{\text{2P}}_{\text{G}}}$$

where *H*_*G*_ is the inertia time constant of the PMSG, *P*_*G*_ is the rated power, and *J* is the moment of inertia.

### DC link capacitor-based virtual inertia control loop

The DC-link capacitor-based virtual inertia control aims to compensate for fluctuations in the output active power and reduce frequency fluctuations. An additional signal is applied to the GSC to provide virtual inertial response by releasing the energy stored in the DC link capacitor Δ*P*_*DC*_. The dynamic equation of the DC-link capacitor is:

11 $$\:{\text{P}}_{{\rm IN\:}}{-\:}{{\rm P}}_{{\rm OUT}}{\:=\:\Delta}{{\rm P}}_{\text{DC}}{\:=}\frac{{\text{C}}_{\text{DC}}{\:}{\text{V}}_{\text{DC}}}{{\text{P}}_{\text{G}}}{\:}\:\text{(}\frac{\text{d}{\text{V}}_{\text{DC}}}{\text{dt}}\text{)}$$

where *C*_*DC*_ is the capacitance of the DC link, *V*_*DC*_ is DC link voltage, *P*_*IN*_ is the input power to DC link capacitor, *P*_*OUT*_is the output from DC link capacitor to GSC. Equating (11) with (8) establishes the link between DC voltage and AC frequency. As a result, the electrostatic potential energy stored in the DC capacitor can provide an inertial contribution to the power system which can be expressed as^13,14^:

12 $$\:{\text{P}}_{{\rm IN\:}}{-\:}{{\rm P}}_{{\rm OUT}}{\:=\:\Delta}{{\rm P}}_{{\rm DC}}{\:}{=\:2\:}\frac{{\text{H}}_{\text{DC}}}{{\text{f}}_{\text{0}}}{\:}\frac{\text{df}}{\text{dt}}{\:=}\frac{{\text{C}}_{\text{DC}}\:{\text{V}}_{\text{DC}}}{{\text{P}}_{\text{G}}}\:\:\text{(}\frac{\text{d}{\text{V}}_{\text{DC}}}{\text{dt}}\text{)}$$

where *H*_*DC*_is the virtual inertia constant of the DC link inertia control. Therefore, by integrating the DC droop control, the relationship between DC voltage and AC frequency is given by^14^:

13 $$\:{\text{C}}_{\text{DC}}{\text{V}}_{\text{DC}}{\:\Delta}{{\:\rm V}}_{\text{DC}}^{{\:\:}}{\:=\:}{{2\:H}}_{\text{DC}}{{\:\rm f}}_{{0}}{\rm \:\Delta\:f}$$

14 $$\:{\text{V}}_{{\rm \:DC,Ref}}{\:=}{{\rm \:V}}_{\text{DC0}}^{{\:\:}}\text{+}\frac{{{2\:H}}_{\text{DC}}{\:\:}{\text{P}}_{\text{G}}}{{{\rm \:V}}_{\text{DC0}}^{{\:\:}}{{\rm \:C}}_{\text{DC}}{{\rm \:\:f}}_{{0}}^{{\:\:\:}}}{\rm \:\Delta\:f\:=}{{\rm \:V}}_{\text{DC0}}^{{\:\:}}\text{+}{\text{K}}_{\text{DC}}{\:\Delta\:f\:\:}$$

where *V*_*DC, Ref*_ is the reference signal for the DC link voltage, *V*_*DC0*_ is the nominal DC link voltage, *K*_*DC*_is the DC Droop constant of the DC link inertia control loop. The virtual inertia constant resulting from the inertial control loop of the DC link can be expressed by^14^:

15 $$\:{\text{H}}_{\text{DC}}{=}\frac{{\text{C}}_{\text{DC}}{{\rm \:f}}_{{0}}{{\rm \:V}}_{{\rm DC0\:}}^{{\:\:2}}}{{4}{{\rm \:P}}_{{\rm G}}{\:\Delta\:f}}{\:[(}\frac{{\Delta}{{\rm \:V}}_{\text{DC}}^{{\:\:}}}{{{\:V}}_{{\rm DC0}}^{{\:\:}}}\text{+1}{\text{)}}^{{\:}\text{2}}\text{-1]}$$

The DC link voltage is allowed to fluctuate within a narrow range, which is set at ± 0.05 pu in this study, while the variation in the system frequency is limited at 0.016pu. The DC-link voltage is kept constant when the WTG is operating in a steady condition. The larger the DC capacitance, the smaller the rate of change of the DC link voltage, from Eq. (15), it is noted that the virtual inertia *H*_*DC*_is limited^15^. With the aid of VCC strategy, which is implemented in the MSC, the output active power can be rabidly changed as the DC link voltage fluctuates, allowing the capacitor to provide a larger contribution of virtual inertia during disturbances The virtual capacitance *C*_*vir*_ can be changed to set the virtual inertia constant. The active power provided using the VCC strategy, ∆*P*_*VCC*_, from the DC link voltage variation can be expressed as^14,15^:

16 $$\:{\Delta}{\text{P}}_{\text{VCC}}{\:=}\:{\text{C}}_{\text{Vir}}{\:}{\text{V}}_{\text{DC}}\frac{{\text{dV}}_{\text{DC}}}{\text{dt}}$$

Differentiating (14) results in

17 $$\:\frac{{\text{dV}}_{\text{DC}}}{\text{dt}}{=}\:\frac{{{\rm 2\:H}}_{{DC}}{\:\:}{\text{P}}_{\text{G}}}{{{\rm \:V}}_{\text{DC0}}^{{\:\:}}{{\rm \:C}}_{\text{DC}}{{\:\:f}}_{\text{0}}^{{\:\:\:}}}{\:\:}\frac{\text{df}}{\text{dt}}\:$$

Substituting (17) in (16),

18 $$\:{{\rm \Delta\:P}}_{\text{VCC}\:}{=}\frac{{{2\:}{\text{C}}_{\text{Vir}}{\rm \:H}}_{\text{DC}}{{\:\rm V}}_{\text{DC}}^{{\:\:}}{\:}{\text{P}}_{\text{G}}}{{{\rm \:V}}_{\text{DC0}}^{{\:\:}}{{\rm \:C}}_{\text{DC}}{{\rm \:\:f}}_{{0}}^{{\:\:\:}}}{\:}\frac{\text{df}}{\text{dt}}{\:}$$

Since the DC link voltage changes within a limited range, it can be assumed that *V*_*DC*_ ≈ *V*_*DC0 .*_ Hence, Eq. (18) can be simplified to

19 $$\:{{\Delta\:P}}_{{\rm VCC\:}}\text{=}\frac{{{2\:}{\text{C}}_{\text{Vir}}{\rm \:H}}_{\text{DC}}{\:}{\text{P}}_{\text{G}}}{{{\rm \:C}}_{\text{DC}}{{\rm \:\:f}}_{{0}}^{{\:\:\:}}}{\:}\frac{\text{df}}{\text{dt}}{\:\:}$$

Comparing (19) with (9), the virtual inertia constant of the VCC control loop, *H*_*vir*_, can be defined as

20 $$\:{\text{H}}_{{\rm Vir\:}}{=}\frac{{{{\rm \:C}}_{\text{Vir}}{\rm \:H}}_{\text{DC}}{\:}}{{{\rm \:C}}_{\text{DC}}}{\:}$$

### **Summary of the control problem**

- Based on an intensive literature review, it has been reported that using fixed virtual inertia gains of the conventional frequency regulation controller prevents the system from providing adequate performance against load disturbance and system parameter variations.
- Owing to the useful features offered by the fuzzy logic control such as modelling complex system responses without the need for mathematical equations, and its ability to deal with system uncertainties, therefore a fuzzy logic controller appears to be a good candidate to enhance the system inertia, considering the power system variations.
- To fill in this gap, this paper presents a hybrid adaptive virtual inertia control strategy based on fuzzy logic to fully utilize the stored kinetic energy and the stored electric energy in the DC-link capacitor, to enhance the overall system inertia and hence the frequency dynamics.

## Proposed control strategy

Using constant gains for virtual inertia control loops increases the equivalent system inertia when responds to disturbances regardless of penetration levels of RES. However, conventional virtual inertia controllers are unable to achieve a satisfactory performance for the same disturbance under varying penetration levels of RES^9,^^10,13,14^. If the microgrid is operated with a large amount of virtual inertia value at frequency deviations with low penetration of renewable energy sources, this will lead to an extended settling time for the frequency profile to be stabilized.

**Fig. 2:**
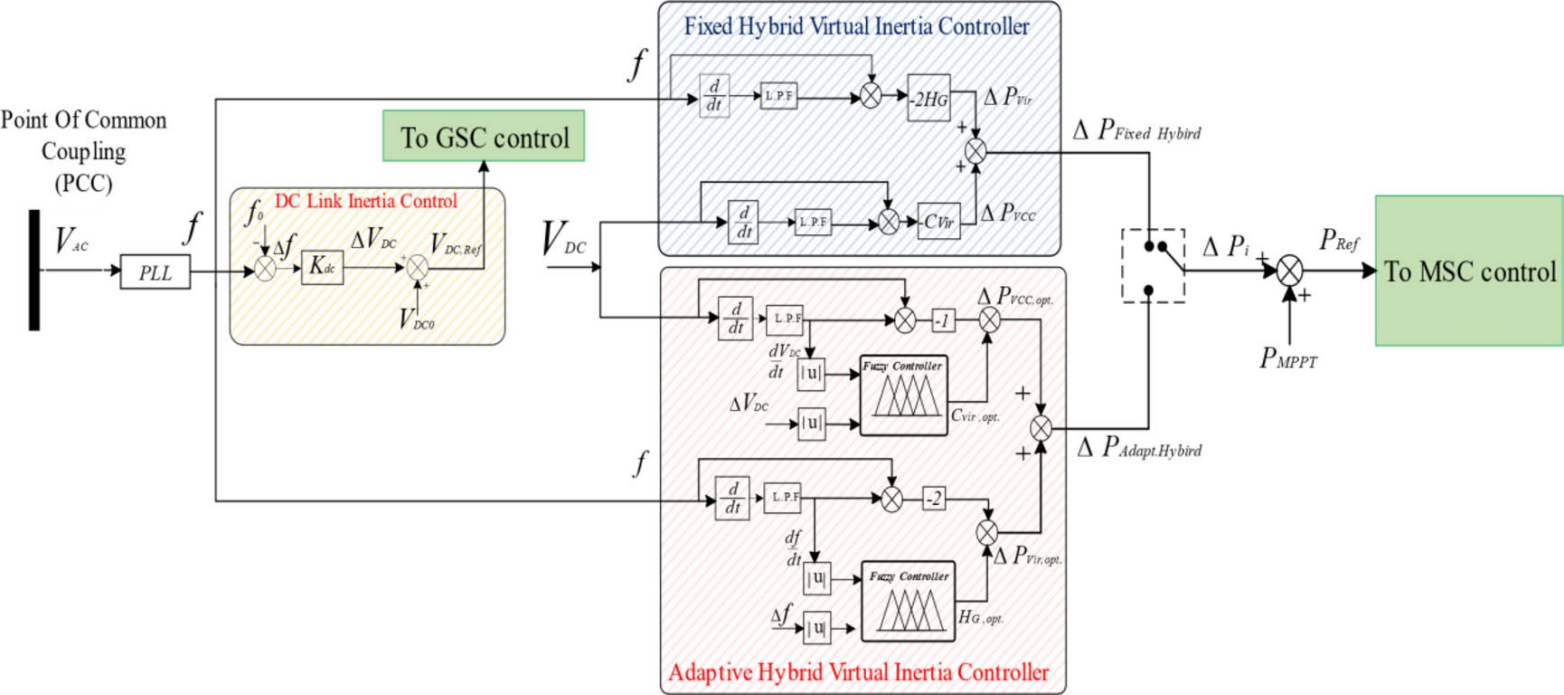
Proposed virtual inertia strategies.

**Fig. 3:**
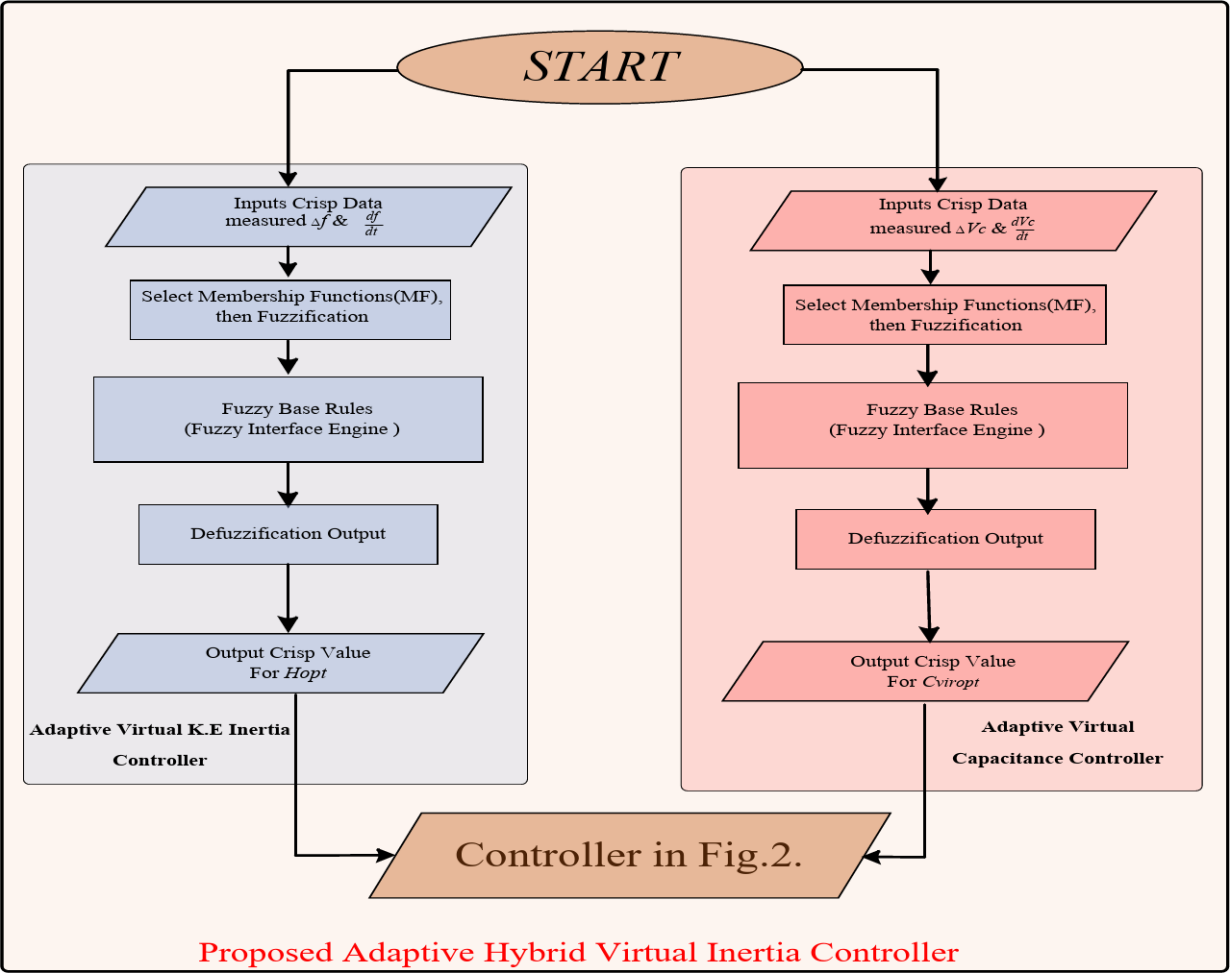
A flow chart for the proposed fuzzy logic controller.

Fuzzy logic has been widely used recently to recover the previous issue by adapting the controller gains. The controller gains are adjusted in response to changes in system performance following the “if-then” compilation rules and fuzzy systems^27–29^. Virtual control loops with adapted gains increase the system inertia and respond to a wide range of RES disturbances and load changes. In this paper, the proposed control strategy combines the KE based virtual inertia control and VCC. Their inertia gains are both adapted using fuzzy logic to improve the dynamic response of the microgrid. Figure 2 illustrates the proposed hybrid virtual inertia controller with fixed and adapted gains. Fuzzy logic is proposed to determine the optimal values of KE-based virtual inertia gain and virtual capacitance. For further clarification, Fig. 3 shows a flow chart of the proposed controller.

**Fig. 4:**
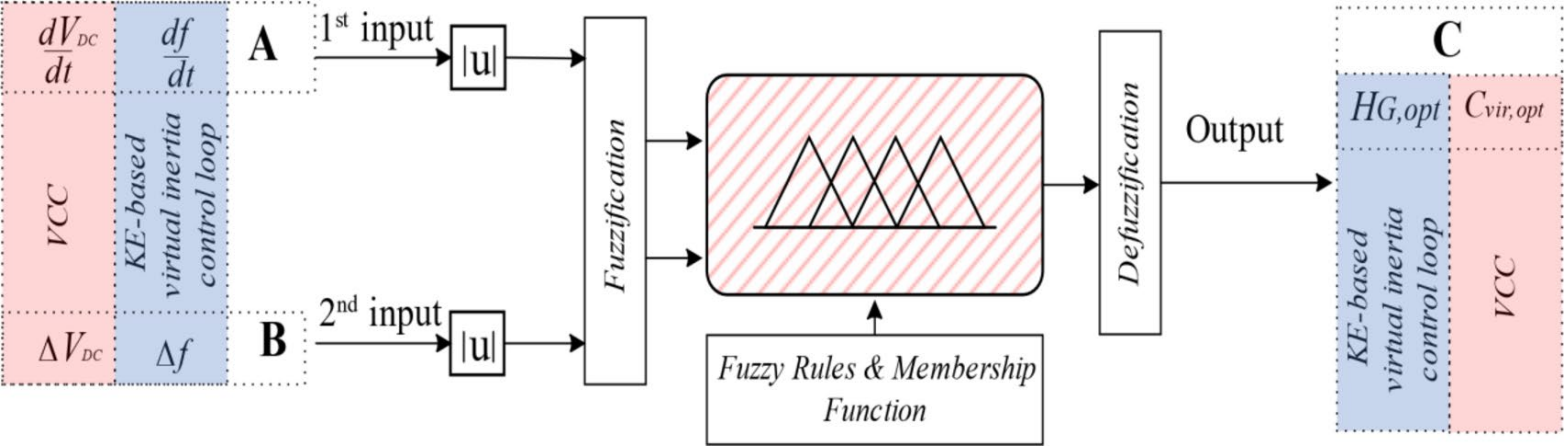
Configuration of the Fuzzy logic system for proposed virtual inertia strategy.

Figure 4 illustrates the configuration of the Fuzzy logic system for proposed virtual inertia strategy. It consists of fuzzy rule base, membership functions, fuzzification, and defuzzification.

The first input A is the signal derivative which is *dV*_*DC*_*/dt* for the adaptive VCC and *df/dt* for the KE based adaptive virtual inertia gain. The second input B is the signal deviation which is *∆V*_*DC*_ for the adaptive VCC and ∆*f* for the KE based adaptive virtual inertia gain. The output C from the first and second Fuzzy logic units are the optimal virtual capacitance *C*_*vir*, opt_ and the optimal virtual inertia gain *H*_*G, opt*_.

**Fig. 5:**
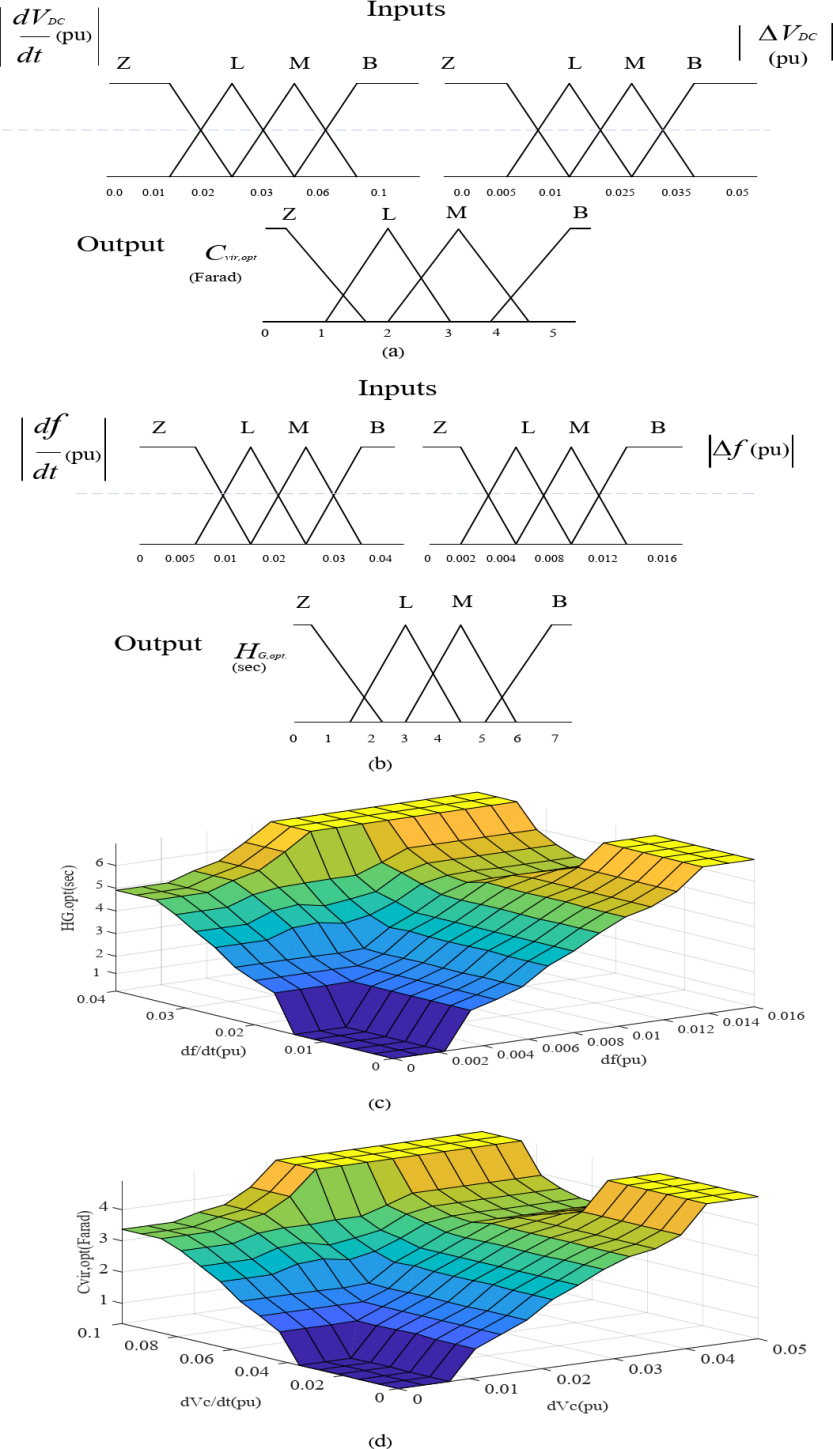
Fuzzy membership functions: (**a**) for adaptive VCC. (**b**) For KE based adaptive virtual inertia. (**c**) Fuzzy surface for adaptive KE based. (**d**) Fuzzy surface for adaptive VCC.

Triangular membership functions are used for the inputs and output as shown in Fig. 5(a) and Fig. 5(b) respectively. The Triangular Membership is selected for this study due to its simple computation and ease of implementation^29,30^. The four linguistic memberships are Z for zero, L for low, M for Medium, and B for Big. For adaptive VCC, the regulation range of ∆*V*_*DC*_ = [0, 0.05] pu. The DC Link Voltage rate of change, *dV*_*DC*_*/dt*, is defined in the range [0, 0.1] pu.

For KE based adaptive virtual controller, the regulation range (∆*f)* = [0, 0.016] pu. The ROCOF, *df/dt*, is defined in the range [0, 0.04] pu to ensure a certain reserve margin. Based on system ratings, the output domains for the gains *H*_*G, opt*_. and *C*_*vir*, opt_ are set to [0,7] Sec and [0,5] Farad, respectively. To determine the optimal gain values for both the VCC and KE-based virtual inertia controller, a fuzzy rule base is used to process fuzzy subsets of linguistic input variables.

Fuzzy rules are based on “IF/THEN” conditional statements and a combined logic of input and output. Sixteen Fuzzy rules are identified in Table 1, which are determined based on the knowledge of virtual inertia control actions and microgrid operation. These rules state that:

-when both the inputs A and B are relatively small, zero membership, Z, is applied for the output C to stabilize the system frequency, delivering fast oscillation damping. The rest of the rules center around assigning medium to large membership to C.
-when the inputs A and B are relatively large, these actions increase the virtual inertia gains to reduce the ROCOF during disturbance, preventing system instability. The rest of the rules center around assigning medium to large membership to C. The fuzzy surfaces for the *H*_*G, opt.*_ and *C*_*vir*, opt_ are shown in Fig. 5(c) and Fig. 5(d), respectively.

**Table 1:** Fuzzy rules.

C (Output) (Optimum Inertia constant)	A 1st input (Signal Derivation)				
		Z	L	M	B
B 2nd input (Signal Deviation)	Z	Z	Z	L	M
	L	L	L	L	B
	M	M	M	M	B
	B	B	M	B	B

Finally, the reference active power *P*_*Ref*_ for the MSC is calculated from:

21 $$\:{\text{P}}_{\text{Ref}}{\:=\:}{\text{P}}_{\text{MPPT}}{\:+\:}{{\rm \Delta\:P}}_{\text{i}}$$

where *∆P*_*i*_ is an additional active power contribution from different frequency support techniques. Table 2. Summarizes the *∆P*_*i*_ settings for different frequency support strategies.

**Table 2:** Various virtual inertia techniques for frequency enhancements.

∆*P*_i_	Case
0, $\:{{\Delta P}}_{\text{0}}\text{=0}$	No Inertia Control
1,$\:{\Delta P}_{1}=2{H}_{G}{f}\:\frac{{\rm df}}{\text{dt}}$	Fixed gain KE-based virtual inertia control
2, $\:{\rm \Delta P}_{\text{2}}\text{=}{\text{C}}_{\text{Vir}}{\text{V}}_{\text{DC}}\:\frac{\text{d}{\text{V}}_{\text{DC}}}{\text{dt}}$	Fixed gain VCC
3, $\:{\Delta P}_{\text{3}}\text{=2}{\text{H}}_{\text{G}}\text{f}\:\frac{\text{df}}{\text{dt}}\text{+}{\text{C}}_{\text{Vir}}{\text{V}}_{\text{DC}}\:\frac{\text{d}{\text{V}}_{\text{DC}}}{\text{dt}}$	Fixed gains hybrid virtual inertia control
4,$\:\:{\Delta P}_{\text{4}}\text{=2}{\text{H}}_{\rm G,\:opt\:\:\:}\text{f}\:\frac{\text{df}}{\text{dt}}\text{+}{\text{C}}_{\rm Vir,\:opt\:}{\text{V}}_{\text{DC}}\:\frac{\text{d}{\text{V}}_{\text{DC}}}{\text{dt}}$	Adaptive gains hybrid virtual inertia control

## Simulation results

To evaluate the effectiveness of the proposed adaptive hybrid virtual inertia controller, the microgrid shown in Fig. 1 is simulated with the help of MATLAB/ Simulink software. The low-inertia grid initially supports a local AC load with 2 MW, whereas the wind turbine injects around 1.15 MW to the microgrid. To compare the performance of the proposed controller with the conventional hybrid virtual inertia controller, different cases studies have been investigated, where the AC load suddenly changes by 10% at time *t* = 15 s under various system parameters variations (wind speed, droop gain, and microgrid inertia). The gains of the hybrid virtual inertia controller are set as follows: *H*_*DC*_= 0.041s. *K*_*DC*_= 58.39, *H*_*vir*_ = 0.75 s, and *C*_*vir*_ = 0.55 F. The parameters of the PMSG-based WT, the low-inertia grid and Case Studies Parameters are listed in Table 3, Table 4, and Table 5, respectively.

**Table 3:** Parameters of the PMSG – based WT.

Parameters	Symbol	Value
PMSG Rated Active Power	*P*_*G*_	1.5 MW
Rated DC Link Voltage	*V*_*DC*_	1150 V
Rated Terminal AC Voltage	*V*_*AC*_	575 V
Rated Wind Speed	*V*_*W*_	12 m/sec
Number of poles pairs	*N*_*P*_	48
Inertia Constant	*H*_*G*_	0.73 s
DC Capacitor	*C*_*DC*_	30 mF
Nominal Frequency	*f*_*0*_	60 Hz
WT Inertia	*J*	35,000 Kg.m2
Rated WT Speed	*ω*_*g*_	7.85 rad/sec

**Table 4:** Parameters of the low inertia gird.

Parameters	Symbol	Value
AC Microgrid Rated Power	*S*_*G*_	2 MW
Turbine Time Constant	*T*_*T*_	0.45 s
Governor Time Constant	*T*_*G*_	0.01 s
Rated Terminal AC Voltage	*V*_*AC*_	575 V
Droop factor	*R*_*eq*_	0.05 pu
Inertia Constant	*H*_*eq*_	1.5 s
Nominal Frequency	*f*_*0*_	60 Hz

**Table 5:** System parameters of case studies.

Case Study	Case
A	*H*_*eq*_=1.5 s., *V*_*W*_=10 m/sec, *R*_*eq*_= 0.05pu
B	*H*_*eq*_=1 s., *V*_*W*_ =10 m/sec, *R*_*eq*_= 0.05pu
C	*H*_*eq*_=1.5 s., *V*_*W*_ =8 m/sec, *R*_*eq*_= 0.05pu
D	*H*_*eq*_=1.5 s., *V*_*W*_=10 m/sec, *R*_*eq*_= 0.033pu

As expected in all case studies, disabling the inertia control results in no change in the rotor speed as the WTG works steadily in MPPT mode and the DC-link is maintained regulated at the nominal setting. In turn, enabling the frequency support loops significantly improved the system frequency response in terms of the frequency nadir and ROCOF. The key findings of the study conducted are summarized in Tables 6 and 7.

**Table 6:** *|ROCOF*_*max*_|, *f*_*nadir*_, and available energy stored results for different case studies.

Simulated Case	Selected Control Strategy	| *ROCOF*_*max*_ | (Hz/sec)	*f*_nadir_ (Hz)	*Available Stored Energy* *(MJ)*	*Energy Released during disturbance* *(MJ)*
Case A Case B Case C Case D	No Inertia Control Fixed Hybrid Virtual inertia control Adapt. Hybrid Virtual inertia control No Inertia Control Fixed Hybrid Virtual inertia control Adapt. Hybrid Virtual inertia control No Inertia Control Fixed Hybrid Virtual inertia control Adapt. Hybrid Virtual inertia control No Inertia Control Fixed Hybrid Virtual inertia control Adapt. Hybrid Virtual inertia control	2.30 1.88 1.56 3.26 2.52 1.96 2.29 1.91 1.55 2.18 1.80 1.51	59.08 59.27 59.35 58.89 59.21 59.3 59.08 59.22 59.41 59.27 59.42 59.52	1.13 1.13 1.13 1.13 1.13 1.13 0.732 0.732 0.732 1.13 1.13 1.13	0 0.01885 0.035 0 0.022 0.04 0 0.016 0.0375 0 0.0167 0.0325

**Fig. 6:**
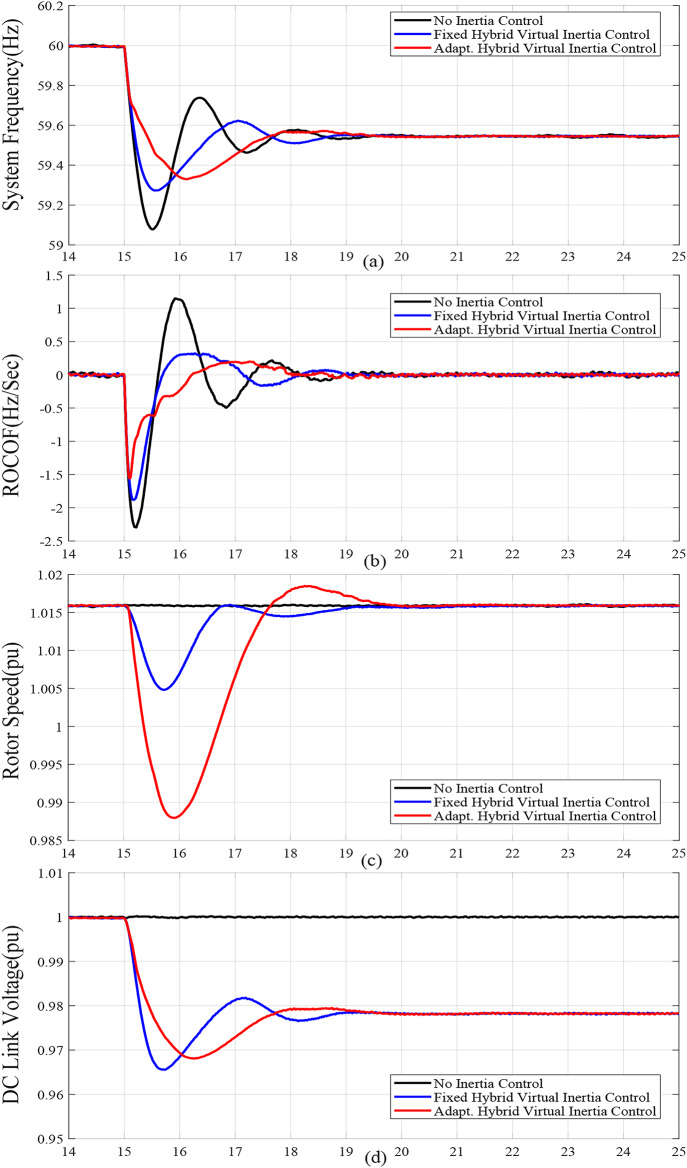

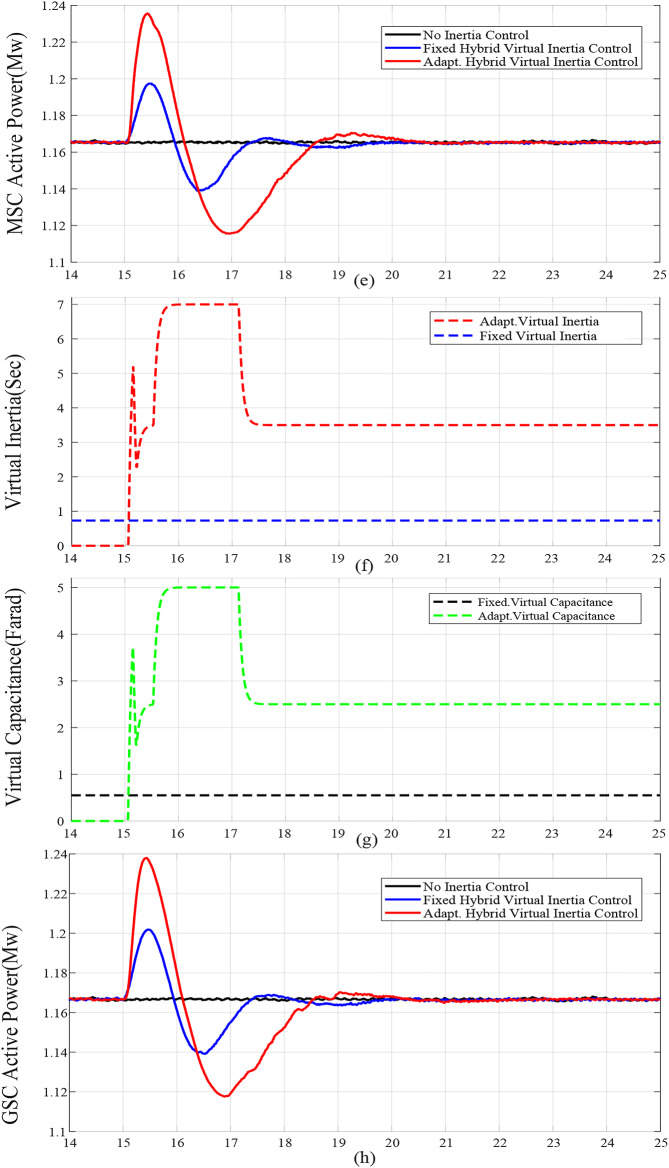
Dynamic response of the proposed system under Case A parameters: (**a**) System Frequency. (**b**) Rated change of System Frequency. (**c**) Rotor Speed Response. (**d**) DC Link Voltage Response. (**e**) MSC Output Active Power. (**f**) Virtual Inertia gain. (**g**) Virtual capacitance gain. (**h**) GSC Output Active Power.

**Fig. 7:**
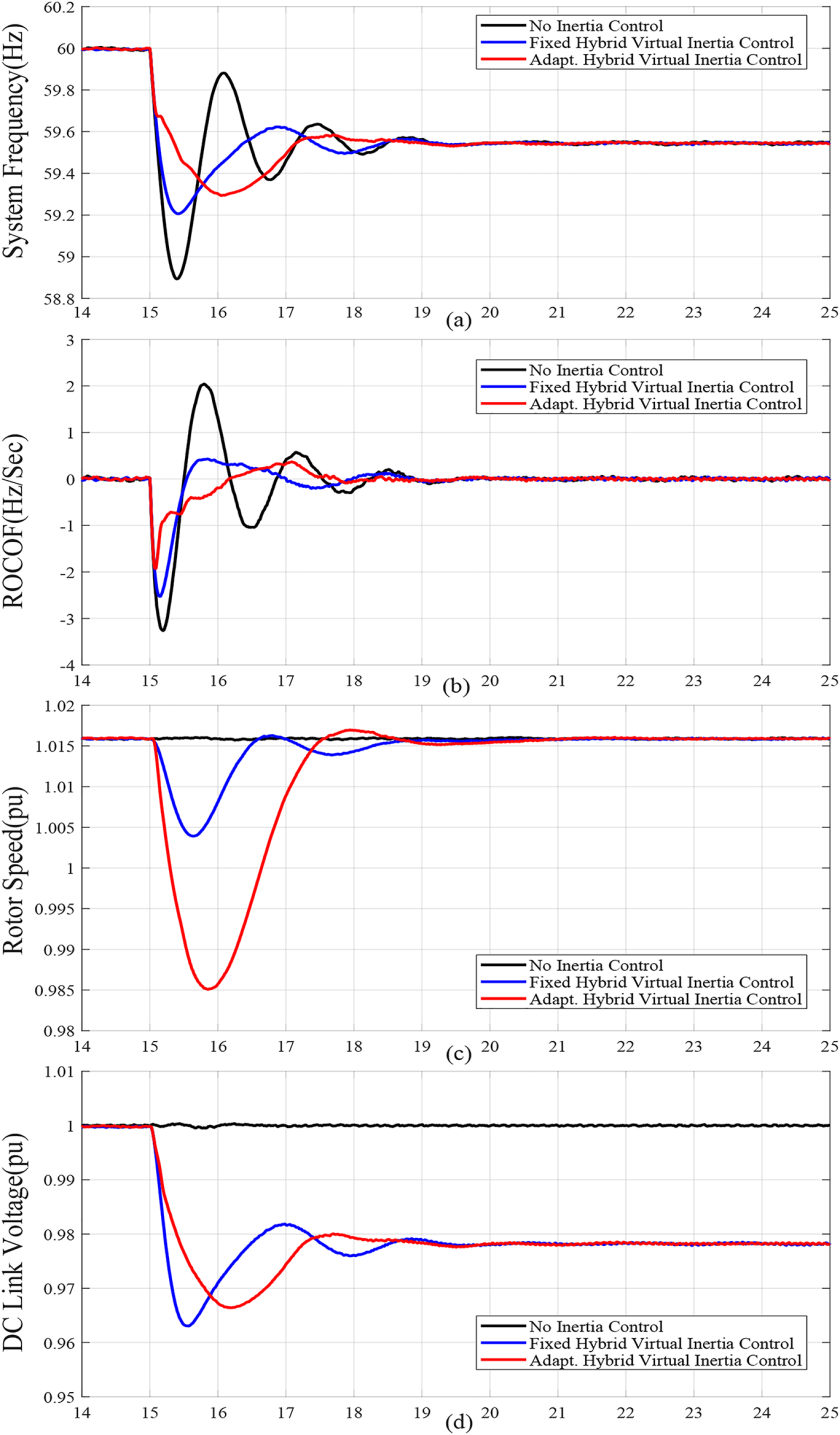

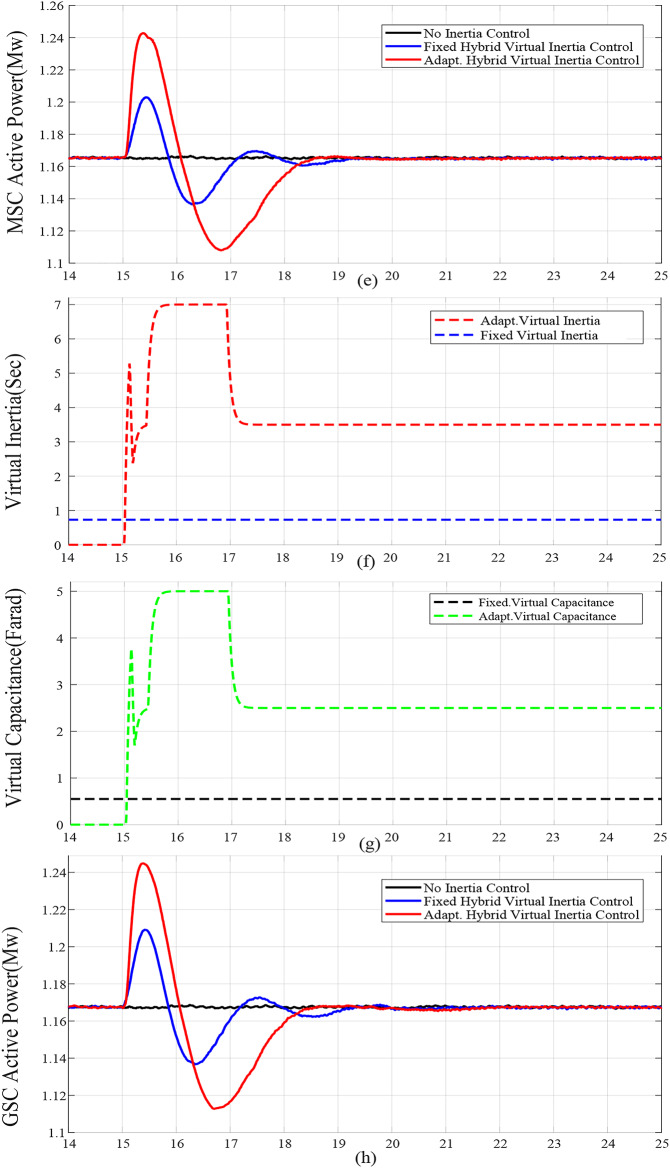
Dynamic response of the proposed system under Case B parameters: (**a**) System Frequency. (**b**)Rated change of System Frequency. (**c**) Rotor Speed Response. (**d**) DC Link Voltage Response. (**e**) MSC Output Active Power. (**f**) Virtual Inertia gain, (**g**) Virtual capacitance gain. (**h**) GSC Output Active Power.

For case studies A and B, the system inertia *H*_*eq*_ is reduced from 1.5s. to 1s., resulting in more fluctuations and increased frequency deviation as illustrated in Fig. 6(a) and Fig. 7(a), respectively. In addition, reducing *H*_*eq*_ reduces the frequency nadir *f*_*nadir*_ as shown in Table 6 regardless of whether the virtual inertia support is activated or not. Compared with the fixed gai hybrid virtual inertia controller, the proposed adaptive hybrid virtual inertia strategy successfully enhances the nadir frequency from 59.27 Hz to 59.35 Hz and from 59.21 Hz to 59.30 Hz for case A and B, respectively. Moreover, the ROCOF is slowed down from 1.88 Hz/s. to 1.56 Hz/s. and from 2.52 Hz/s. to 1.96 Hz/s. for case A and B as shown in Fig. 6(b) and Fig. 7(b), respectively. It is worth noting that the proposed adaptive hybrid virtual inertia strategy provides further performance improvement for low inertia systems. However, the deviation in rotor speed for the proposed adaptive strategy is the highest compared with the fixed gain controller as indicated in Fig. 6(c) and Fig. 7(c). This action results from releasing more power though the MSC when the system inertia is reduced, as demonstrated in Fig. 6(e) and Fig. 7(e), to improve the microgrid dynamic performance.

Furthermore, Fig. 6(d) and Fig. 7(d) illustrate the increasing deviation of the DC link voltage as an action of the DC link droop control loop, integrated with the GSC controller, when the system inertia is decreased.

**Fig. 8:**
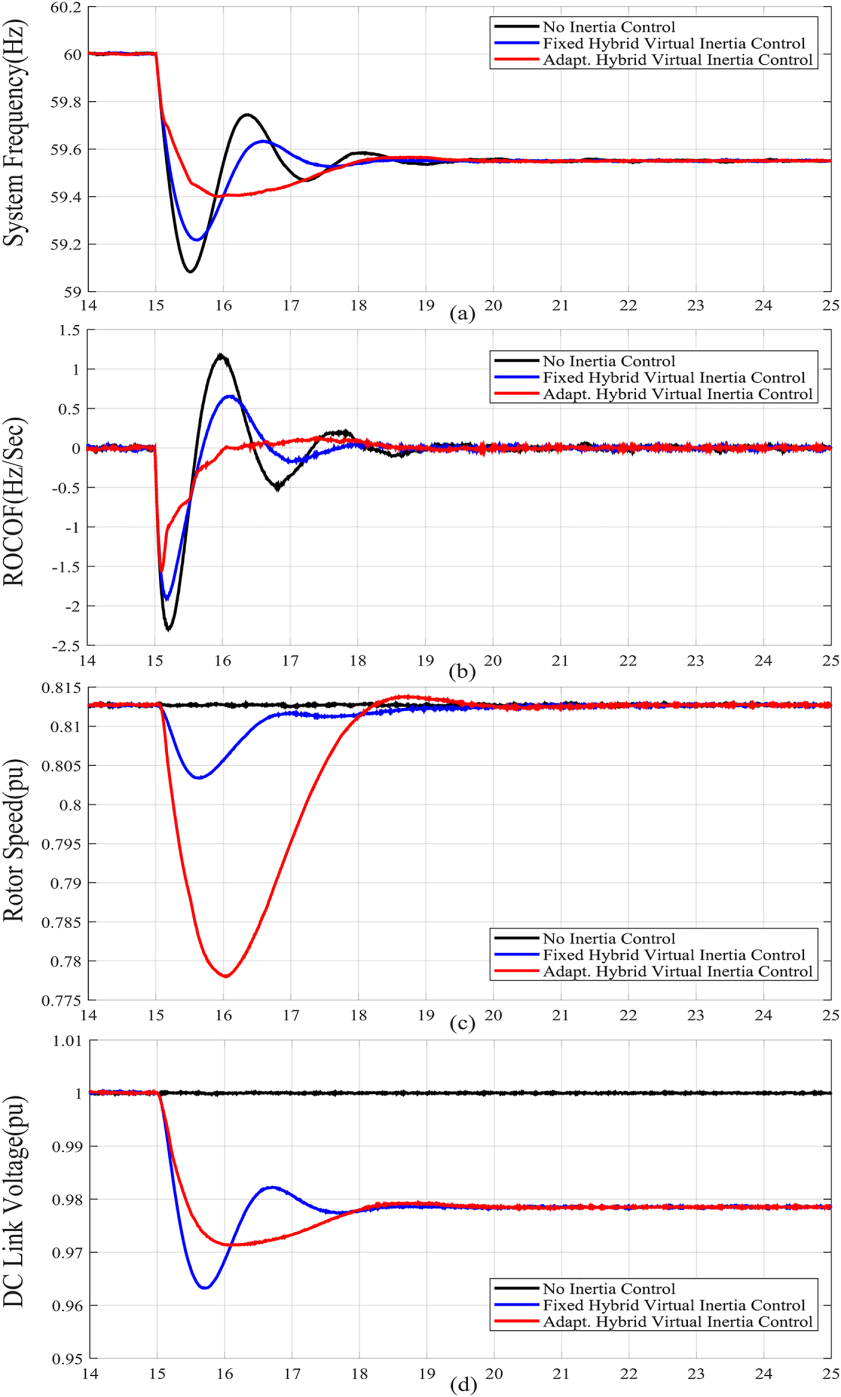

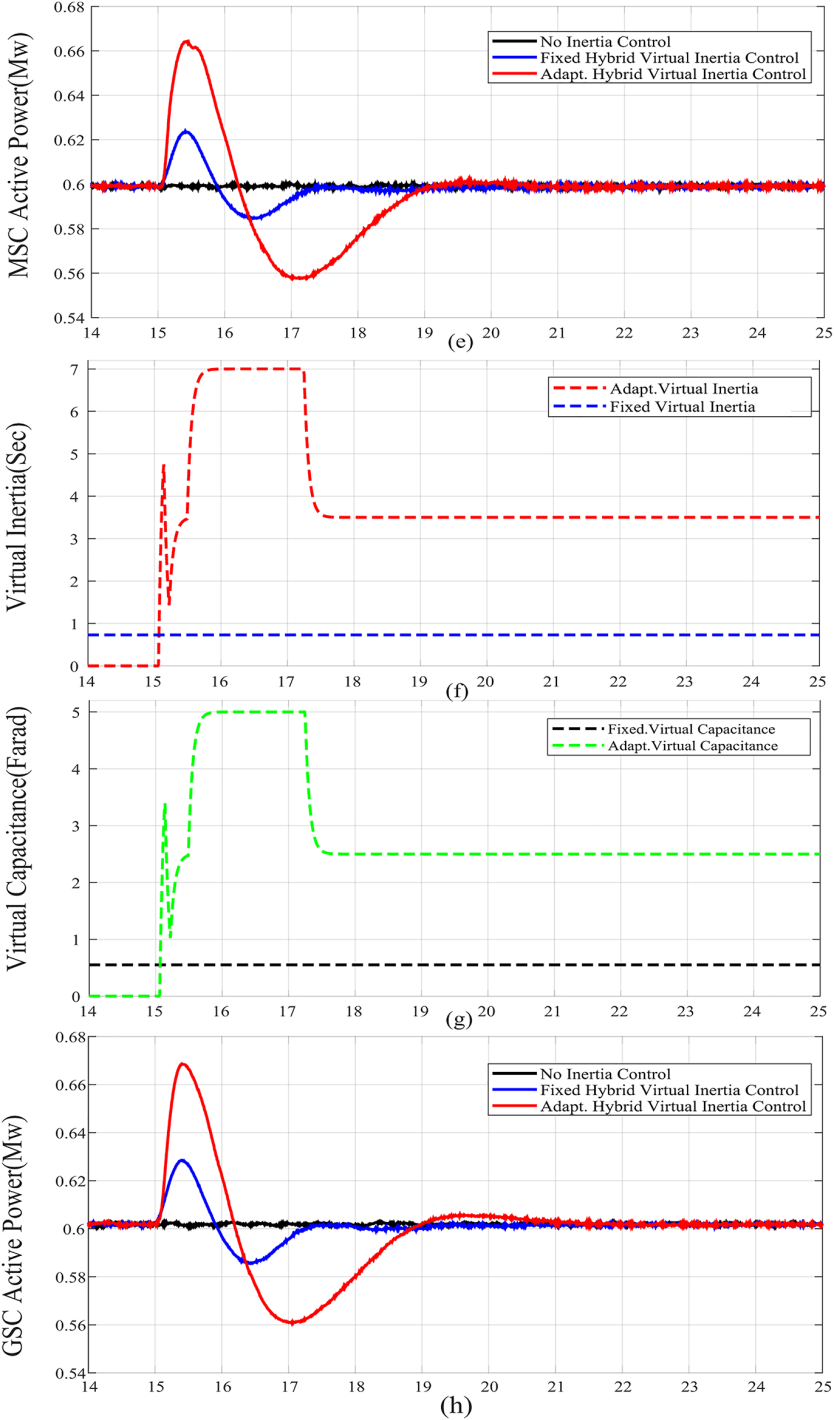
Dynamic response of the proposed system under Case C parameters: (**a**) System Frequency. (**b**)Rated change of System Frequency. (**c**) Rotor Speed Response. (**d**) DC Link Voltage Response. (**e**) MSC Output Active Power. (**f**) Virtual Inertia gain. (**g**) Virtual capacitance gain. (**h**) GSC Output Active Power.

When the wind speed is reduced, case C, the frequency nadir is reduced and the ROCOF has increased as shown from comparing Fig. 8 with Fig. 6. Compared with the fixed gain hybrid virtual inertia controller, the proposed adaptive hybrid virtual inertia strategy successfully enhances the nadir frequency from 59.22 Hz to 59.41 Hz and slows down the ROCOF from 1.91 Hz/s. to 1.55 Hz/s. as illustrated in Fig. 8.

**Fig. 9:**
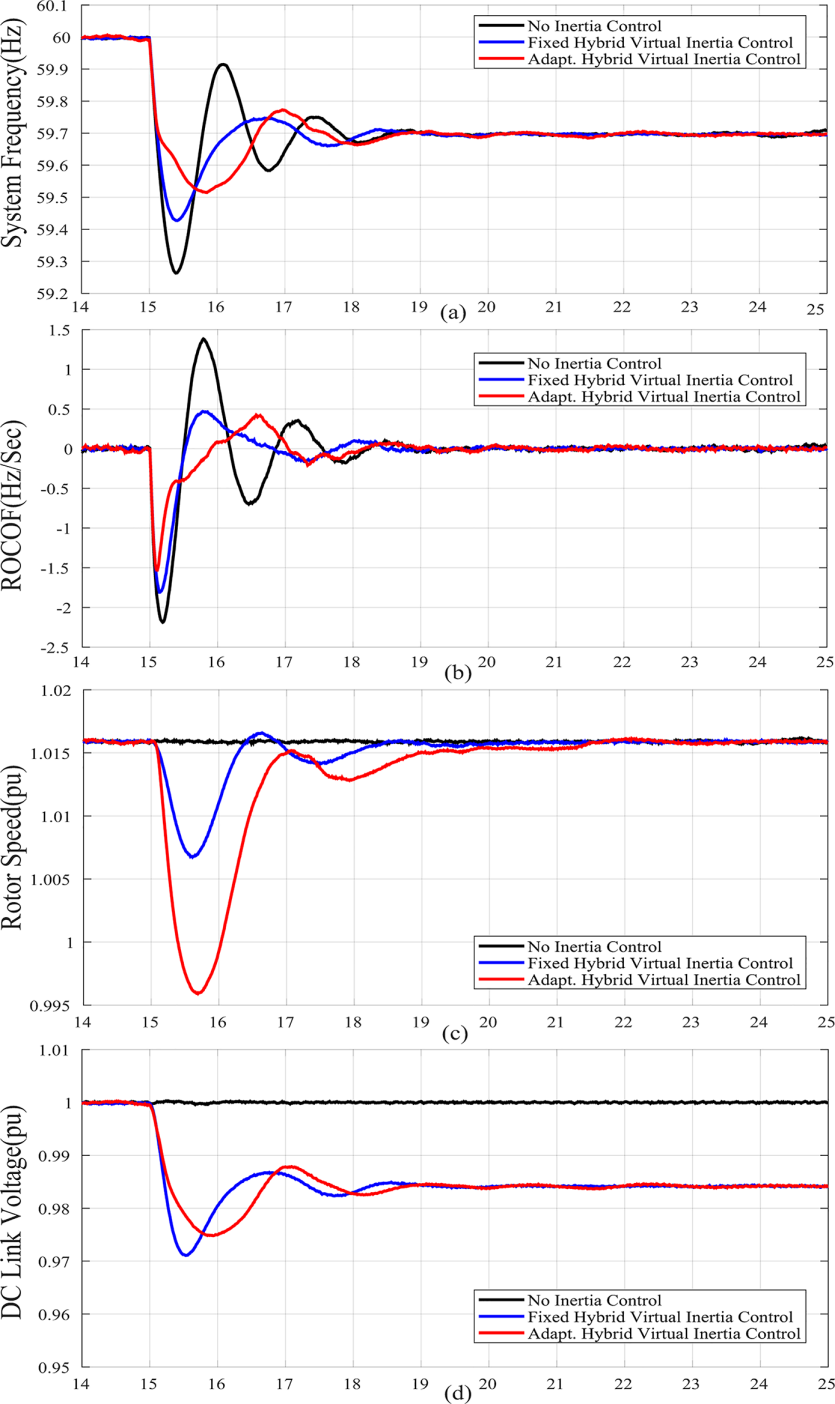

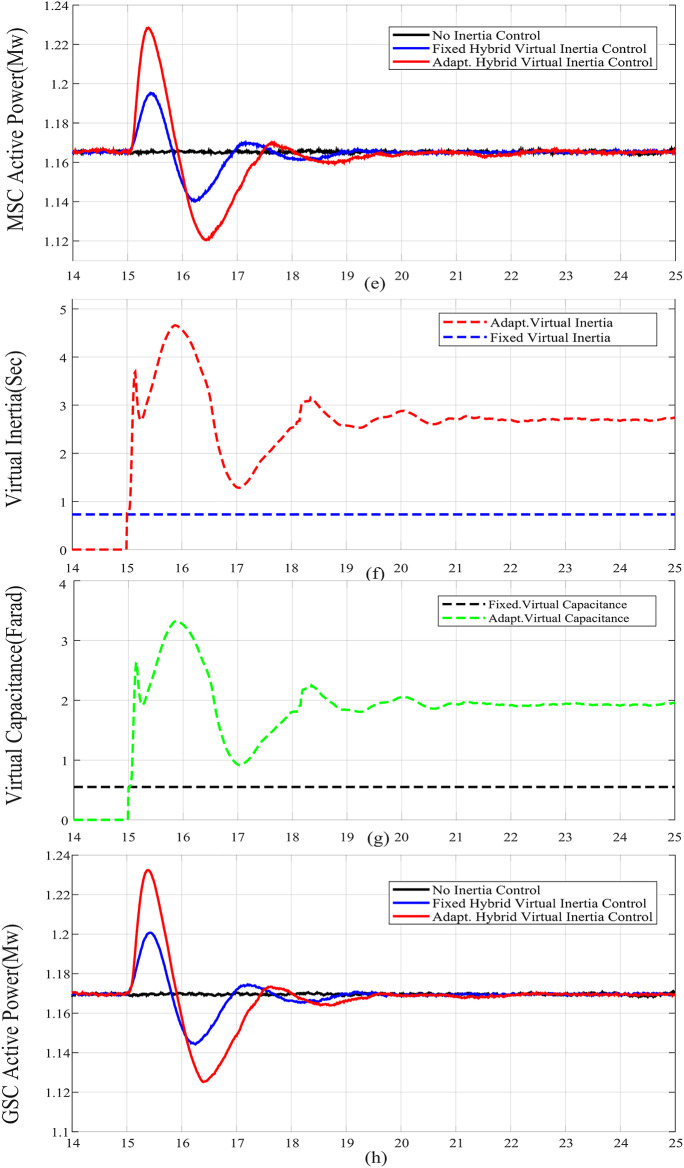
Dynamic response of the proposed system under Case D parameters :(**a**) System Frequency. (**b**)Rated change of System Frequency. (**c**) Rotor Speed Response. (**d**) DC Link Voltage Response. (**e**) MSC Output Active Power. (**f**) Virtual Inertia gain, (**g**) Virtual capacitance gain. (**h**) GSC Output Active Power.

Reducing the SG droop factor, case D, reduces the frequency deviation and ROCOF as shown in Fig. 9 compared to Fig. 6 As a result, the burden on WTG for the frequency support is reduced. Again, the proposed adaptive hybrid virtual inertia strategy outperforms the fixed gain hybrid virtual inertia controller by boosting the nadir frequency from 59.42 Hz to 59.52 Hz and slowing down the ROCOF from 1.8 Hz/s. to 1.51 Hz/s. as illustrated in Fig. 9.

Figure 6(f), Fig. 7(f), Fig. 8(f), Fig. 9(f), Fig. 6(g), Fig. 7(g), Fig. 8(g), and Fig. 9(g) compare the gains of the fixed and the proposed adaptive virtual inertia gains (*H*_*G, opt*_ and *C*_*vir, opt*_*)* for the investigated cases studies. It is obvious that the gains are dynamically adjusted according to the deviation in the frequency, dc-link voltage deviation (∆*f* and ∆*V*_*DC*_) and their rate of change (*df/dt* and *dV*_*DC*_*/dt*). It is worth noting that both gains reach the maximum saturation level for around 2 s for cases A-C, whereas for case D, both gains vary dynamically hitting the maximum values. This is because the active power contribution of the low-inertia grid increases when the droop gain is reduced, yielding in a smaller steady-state frequency and dc voltage deviation. Consequently, this behavior affects the frequency support demand from the wind turbine, resulting in reduced adaptive gains for both steady-state and dynamic(transient) values.

Finally, Fig. 6(h), Fig. 7(h), Fig. 8(h), and Fig. 9(h) show the injected active power to the GSC, where the available stored energy in the wind turbine system (wind generator rotor and the dc-link capacitor) and the energy required to support the low-inertia system at the disturbance event, can be directly calculated. As shown in Table 6, the available stored energy is significantly affected with the wind speed, where case C offers the lowest amount of energy at 8 m/s wind speed, if compared to the other cases at higher wind speed (10 m/s). It is worth mentioning that the wind turbine system provided the AC grid with the largest amount of energy in case B if compared to the other cases. This is because case B represents the lowest inertia of the AC system (*H*_*eq*_= 1 s), therefore, the amount of energy required from the wind generator is the highest. Further, it can be also noted that the maximum amount of energy required to support the low-inertia system is only around 5% of the available stored energy, as demanded in Case C.

**Table 7:** System parameter results for different case studies.

Simulated Case	Selected Control Strategy	*Steady State Frequency Deviation* *∆f*_*s.s*_ *(Hz)*	*Damping Ratio(pu)*	*Frequency oscillation (Hz)*	*Mode of Oscillation*
Case A Case B Case C Case D	No Inertia Control Fixed Hybrid Virtual inertia control Adapt. Hybrid Virtual inertia control No Inertia Control Fixed Hybrid Virtual inertia control Adapt. Hybrid Virtual inertia control No Inertia Control Fixed Hybrid Virtual inertia control Adapt. Hybrid Virtual inertia control No Inertia Control Fixed Hybrid Virtual inertia control Adapt. Hybrid Virtual inertia control	0.458 0.458 0.458 0.458 0.458 0.458 0.458 0.458 0.458 0.3 0.3 0.3	0.225 0.4 0.55 0.2 0.398 0.55 0.25 0.388 0.52 0.228 0.453 0.52	0.573 0.391 0.364 0.718 0.384 0.356 0.572 0.503 0.18 0.728 0.45 0.46	Under Damped Under Damped Under Damped Under Damped Under Damped Under Damped Under Damped Under Damped Under Damped Under Damped Under Damped Under Damped

Looking at the system stability parameters, Table 7 shows different system’s mathematical quantities, such as the mode of oscillations, damping ratio, frequency oscillation, and steady-state frequency deviation. It is clear that for case studies A, B, C and D, the proposed hybrid adaptive controller is able to enhance the system damping, resulting in improving the damping ratio from around its lowest value 0.2 pu (Case B*)* to around 0.55 pu as in Cases A and B, and 0.52 pu as in Cases C and D. It should be noted that steady-state frequency deviation is the minimum in Case D, this is because the power sharing droop gain is lower in Case D, which allows the synchronous generators in the low-inertia system to inject more power during a load demand increase, yielding a lower steady-state frequency deviation.

### Remark

It should be pointed out that although the proposed controller showed remarkable improvements in the system dynamics. However, the fuzzy logic controllers suffer from the absence of clear design guidelines if compared with traditional controllers. Therefore, the design process often relies on the designer’s experience and deep knowledge about the system, making it difficult to ensure that a controller will meet the desired performance objectives. The absence of established guidelines can introduce uncertainty and increase the complexity of controlling development. Nevertheless, the Mamdani fuzzy inference system controller proposed in this work can be extended by employing the Adaptive Network Fuzzy Logic Control (ANFIC). The ANFIC is more trained and more adaptive than Mamdani fuzzy inference system, this controller is expected to further improve and optimize the overall system dynamics through developing newer and better membership functions.

## Conclusion

This paper proposes a hybrid adaptive virtual inertia controller based on fuzzy logic. The presented strategy utilizes the mechanical energy stored in the wind turbine rotor and the energy of the DC-link capacitor to improve the frequency behavior of low-inertia grids in terms of the frequency nadir and rate of change with time. The proposed controller processes the rate of change and the deviation of both the frequency and dc-link voltage signals via two fuzzy logic units to adaptively generate the gains of virtual inertia and the virtual capacitor control loops. Four case studies considering the uncertainty in the system parameters have been presented in this work to assess and evaluate the proposed controller performance under different operating conditions and parameters. The proposed controller’s performance is compared with the case where no frequency support is available and when controller with fixed gains is used. It has been shown that the system performance with the adaptive controller outperforms that with fixed gains even at different operating conditions and system parameters. The simulation obtained results justify the effectiveness of the proposed controller.

## Data Availability

The datasets used and/or analysed during the current study available from the corresponding author on reasonable request.
